# Recent progress in thyroid surgery

**DOI:** 10.1186/1756-6614-6-S2-A15

**Published:** 2013-04-05

**Authors:** Marek Dedecjus

**Affiliations:** 1 Department of General, Oncological and Endocrine Surgery, Medical University of Lodz, Poland

## 

The management of thyroid disease has evolved rapidly within the past decade. The history of thyroid surgery starts in the second half of XIX century with Billroth, Kocher and Halsted, who developed safe technique of thyroid resection. Since then, the objectives for thyroidectomy are: conservation of the parathyroid glands and preserving laryngeal nerves, an accurate hemostasis and recently excellent cosmesis.

In the recent decades, the dynamic development of new technologies, applied in thyroid surgery caused major improvements in results of this type of surgery. In particular, a large progress in minimally-invasive thyroidectomy, new dissecting devices and intraoperative neuromonitoring have been observed. Moreover, recent progress in robotic surgery and transoral thyroidectomy indicates new fields of development of thyroid surgery. In addition, radiological advances are changing the way in which we manage thyroid diseases and continue to aid in the diagnosis and treatment of thyroid cancer. New imaging techniques have been implemented to facilitate the diagnosis and management of thyroid cancer by multidisciplinary teams. Apart from surgical techniques, our understanding of the molecular genetics of thyroid diseases continues to progress, and with it new techniques for diagnosis and treatment are expected to develop.

